# Metabolites from *Bacillus subtilis* J-15 Affect Seedling Growth of *Arabidopsis thaliana* and Cotton Plants

**DOI:** 10.3390/plants11233205

**Published:** 2022-11-23

**Authors:** Hui Zhang, Qilin Yang, Jingjing Zhao, Jiayi Chen, Shiqi Wang, Mingyue Ma, Huan Liu, Qi Zhang, Heping Zhao, Dongyuan Zhou, Xianxian Wang, Jie Gao, Huixin Zhao

**Affiliations:** 1 Xinjiang Key Laboratory of Special Species Conservation and Regulatory Biology, College of Life Science, Xinjiang Normal University, Urumqi 830054, China; 2 Beijing Key Laboratory of Gene Resource and Molecular Development, College of Life Sciences, Beijing Normal University, Beijing 100875, China

**Keywords:** *Bacillus subtilis* J-15, LC-MS, plant growth regulation, hormone signaling pathways

## Abstract

*Bacillus subtilis* J-15 is a plant growth-promoting rhizobacteria isolated from the soil rhizosphere of cotton and is resistant to cotton verticillium wilt. This study evaluated the effects of metabolites of J-15 (J-15-Ms), including mycosubtilin, on plant growth using *Arabidopsis* and cotton plants. The results showed that J-15-Ms promoted *Arabidopsis* seeding growth at lower concentrations of 0.2 μg/mL but inhibited the growth at higher concentrations, such as 20 μg/mL. Similar results were obtained in cotton. Thus, J-15-Ms-treated plants showed low-concentration-induced growth promotion and high-concentration-induced growth inhibition. The J-15-Ms components were analyzed by liquid chromatography–mass spectrometry. Correlation analysis using the J-15 genomic databases suggested that J-15 may synthesize indoleacetic acid via the indole-3-pymvate pathway and indole-3-acetamide pathway. Treatment with mycosubtilin, a purified peptide from J-15-Ms, showed that the peptide promoted Arabidopsis growth at a low concentration (0.1 μg/mL) and inhibited plant growth at high concentrations (higher than 1 μg/mL), which also significantly increased plant lateral root number. Transcriptomic analysis showed that mycosubtilin might promote lateral root development and inhibit plant primary root growth by regulating the expression of the plant hormone signaling pathway. This study reveals the mechanism of *Bacillus subtilis* J-15 in affecting plant growth.

## 1. Introduction

Plant rhizosphere soils contain various microorganisms such as bacteria, fungi, actinomycetes, and soil protozoa. These microorganisms include not only pathogenic bacteria that cause damage to plants, but also probiotics that promote host plant growth, nutrient absorption, resistance to stress, and pathogenic bacteria [1]. Among them, the beneficial bacteria that colonize plant rhizosphere and promote plant growth are known as PGPR [2]. PGPR colonizes the plant root system and promotes active plant growth through direct mechanisms, such as nutrient solubilization, nitrogen fixation, and growth production [3], or indirect mechanisms, such as root development stimulation, competitive exclusion of pathogens, or removal of phytotoxic substances [4]. Various PGPR strains, including *Agrobacterium*, *Bacillus*, *Burkholderia*, *Pseudomonas*, and *Serratia*, have been identified [5]. These PGPRs promote plant growth, inhibit pathogenic bacteria, and maintain the ecological balance of the root system by regulating the microenvironment of plant growth and development [6]. Root inoculation with *Azotobacter chroococcum* 76A enhanced the nutrient assimilation efficiency and promoted the growth of tomato plants under salt stress [7]. *Bacillus subtilis* strain GOT9 enhanced tolerance against drought and salt stresses and improved lateral root growth in *Arabidopsis* [8]. The rhizobacterial strains BKM20 and BKM04 also promoted *Lolium perenne* L. growth and improved soil fertility and microbial activity [9]. *Pseudomonas* sp. P8, *Peribacillus* sp. P10, and *Streptomyces* sp. X52 promoted growth and improved the adaptability of maize to salt stress [10]. Furthermore, *Bacillus amyloliquefaciens* B14 reduced the charcoal root rot incidences in common peas [11]. *Enterobacter* 64S1 and *Pseudomonas* 42P4 isolated from tomato roots showed strong phosphate solubilizing activity and promoted the growth of tomato seedlings with reduced fertilizer application [12].

Among the PGPRs, *Bacillus* is the most studied due to its ease of isolation from soil and plants, broad metabolite profile, rapid growth, and ability to colonize plant surfaces [13]. *Bacillus* can exert its growth-promoting activities on plants directly by producing plant growth regulators, such as growth hormones, cytokinins, and gibberellins [14], or through nitrogen fixation, phosphate solubilization, and ironophilins production [15]. Most *Bacillus* species can also synthesize and release indoleacetic acid (IAA) [16]; for example, *Bacillus* sp. UCMB5113 can produce cytokinin and indole-3-acetic, affecting plant root growth [17]. *Bacillus strain* J119 produces IAA and iron carriers to promote the growth of oilseed rape, maize, tomato, and other plants [18]. *Bacillus megaterium* BP17 promotes *Arabidopsis* growth by downregulating ethylene-responsive genes and upregulating nutrient absorption-related genes [19]. Inoculation of *Bacillus subtilis* strain L1 promoted nitrate utilization and plant growth [20]. Furthermore, *Bacillus* HNH7 and HNH9 reportedly promote the growth of land cotton by producing iron carriers to dissolve iron and upregulate cotton growth-related genes [21]. *Bacillus aryabhattai* LAD exhibits phosphate solubilization and nitrogen fixation activities, which facilitate root development and growth of maize seedlings [22]. *Bacillus subtilis* EA-CB0575 also promotes tomato plant growth by dissolving phosphorus, repairing nitrogen, and producing indole and iron cell-containing compounds [23]. The volatile compounds excreted by *Bacillus subtilis* also play an important role in regulating plant growth [24]. The volatiles emitted by *Bacillus subtilis* (GB03) have a lasting beneficial effect on the growth of *Arabidopsis* [25]. 

In addition to hormones, *Bacillus* also stimulates plant growth indirectly via indirect mechanisms by producing 1-aminocyclopropane-1-carboxylate (ACC) deaminases, antibiotics, cell wall-degrading enzymes, and hydrogen cyanide, as well as by activating host systemic resistance and ferrocarriers [6]. For example, *Bacillus subtilis* Rhizo SF 48 produces ACC deaminase, which promotes plant growth and induces drought resistance in tomatoes [26]. The beneficial soil bacterium *Bacillus subtilis* (GB03) treatment enhanced Arabidopsis Choline synthesis and improved plant tolerance to osmotic stress [27]. *Bacillus subtilis* 5YN8 and DSN012 have high antagonistic and hydrolytic enzyme activities, which significantly inhibit pepper grey mold and promote pepper growth [28]. Moreover, *Bacillus subtilis* CBR05 can directly inhibit the growth of pathogens and improve pathogen resistance by enhancing the systemic resistance in tomatoes [29]. Many *Bacillus* species have been reported to produce various antimicrobial compounds from the lipopeptide family [30], including surfactins, iturins, and fengycins, which have strong antagonistic activity against various plant pathogenic fungi [31,32,33]. *Bacillus* Fcl1 produces a mixture of iturin A and surfactants that act as antifungal agents [34]. *Bacillus subtilis* 6051 is able to form biofilm on roots and secrete surfactin to protect plants from pathogenic bacteria [35]. *Bacillus subtilis* SG_JW.03 triggers systemic resistance in plants by secreting antifungal lipopeptides to suppress pathogens and upregulate host plant pathogenesis-related genes [36]. The cyclic lipopeptides (CLPs) of *Bacillus subtilis* ABS-S14 can activate the plant defense pathways, improve resistance, and inhibit chloromycnosis caused by citrus penicillium [37]. *Bacillus subtilis* M4 also showed the potential to protect plants from fungal diseases, such as bean sprout wilt and apple gray mold caused by putrescence, via different pathogenic systems through its secreted fungicides [38]. Moreover, mycosubtilin and surfactin secreted by *Bacillus subtilis* can induce resistance against pathogenic fungal spores in grapes [39]. Despite these disease-resistance activities, the effects of lipopeptides on plant growth are not well studied.

*Bacillus subtilis* J-15, a probiotic strain isolated from the inter-rhizosphere soil of cotton, produces metabolites (J-15-Ms) that can effectively inhibit the infection by *Verticillium dahliae*, thus reducing the incidence of verticillium wilt in cotton [40]. J-15-Ms have broad-spectrum resistance to various plant pathogens [41] and have no deleterious effects on the abundance and diversity of soil microbial communities [42]. However, whether the J-15-Ms regulate plant growth and development is unknown. This study investigated the effects of J-15-Ms on the growth and development of *Arabidopsis* and cotton seedlings. The J-15-Ms metabolites were analyzed by LC-MS. The previous analysis of the J-15 genome found that the major antifungal gene clusters reported in *Bacillus subtilis* only contained the complete mycosubtilin and bacilibactin manipulators, suggesting that the two could be the major antifungal active substances [43]. Another study also isolated and purified the J-15-Ms and identified the antagonist as mycosubtilin [44]. Based on this, we investigate the mycosubtilin plant growth regulatory mechanism and provide a theoretical basis for applying the J-15 strain in plant growth improvement.

## 2. Results

### 2.1. Effect of J-15-Ms on Arabidopsis Growth

**Figure 1:**
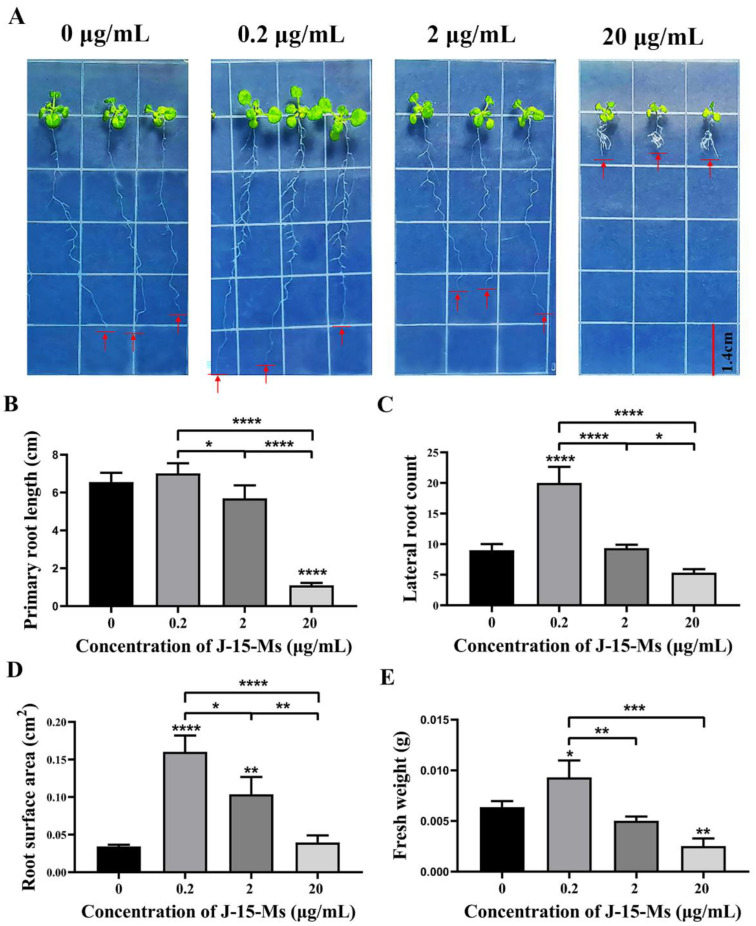
Effect of J-15-Ms on the growth of *Arabidopsis* seedlings. (**A**) Arabidopsis seedling growth phenotype. (**B**) Effect on primary root length. (**C**) Effect on lateral root number. (**D**) Effect on root surface area. (**E**) Effect on fresh weight. Schemes follow the same formatting. Note: Means ± SEs, n = 50, different “*” indicate significant differences among treatments based on the least significant difference at *p* ≤ 0.05.

After treating *Arabidopsis* seedlings with J-15-Ms for 10 days, we found that *Arabidopsis* growth was promoted at lower J-15-Ms concentrations but inhibited at higher concentrations. At 0.2 μg/mL, the J-15-Ms promoted the growth of *Arabidopsis* primary roots, significantly increasing their root surface area and fresh weight and the number of lateral. However, when the concentration was increased to 2 μg/mL, J-15-Ms inhibited the growth of *Arabidopsis* primary roots and the development of *Arabidopsis* lateral root (Figure 1). These findings suggest that J-15-Ms acted in a concentration-dependent manner; the higher the concentration, the more the inhibition of *Arabidopsis* primary root, an effect that was more obvious at 20 μg/mL. This suggests the potential of J-15-Ms in controlling plant growth by regulating plant root development.

### 2.2. Effect of J-15-Ms on Cotton Growth

**Figure 2:**
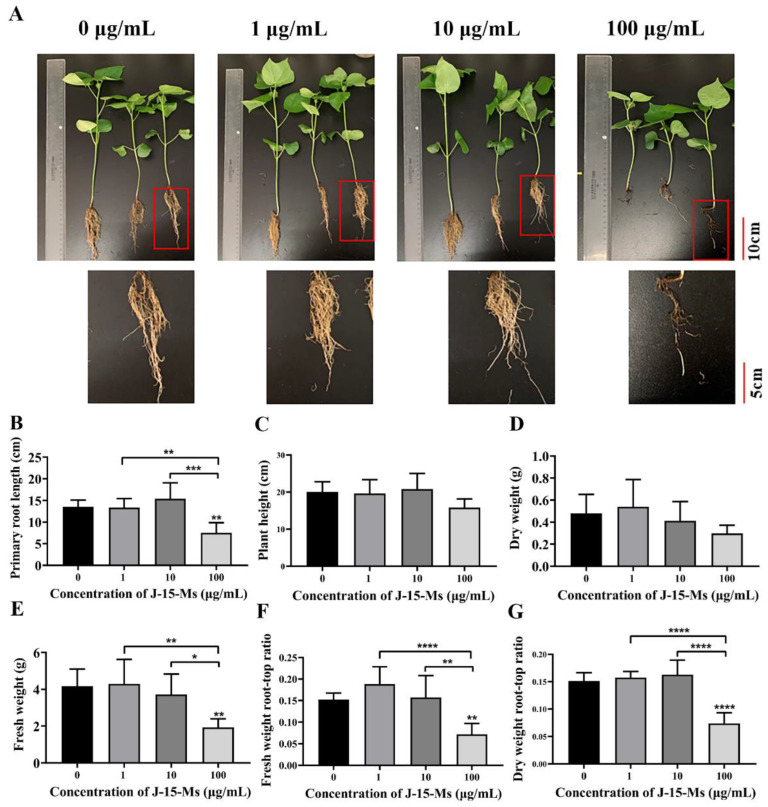
Effects of J-15-Ms on the growth of cotton seedlings. (**A**) Cotton seedling growth phenotype. (**B**) Effect on primary root length. (**C**) Effect on plant height. (**D**) Effect on dry weight. (**E**) Effect on fresh weight. (**F**) Effect on fresh weight root-top ratio. (**G**) Effect on dry weight root-top ratio. Note: Means ± SEs, n = 50, different “*” indicate significant differences among treatments based on the least significant difference at *p* ≤ 0.05.

After 21 days of treating cotton seedlings with different J-15-Ms concentrations, the J-15-Ms at 1 μg/mL increased the fresh weight and dry weight as well as the fresh weight root-to-crown ratio in cotton. At 10 μg/mL, J-15-Ms increased the plant height, root length, and dry weight root-to-crown ratio in cotton; however, the growth of cotton was significantly inhibited at J-15-Ms concentration of 100 μg/mL (Figure 2). Thus, similar to *Arabidopsis*, J-15-Ms promoted cotton growth at lower concentrations but inhibited it at higher concentrations.

### 2.3. LC-MS Identification and Taxonomic Analysis of J-15-Ms

**Figure 3:**
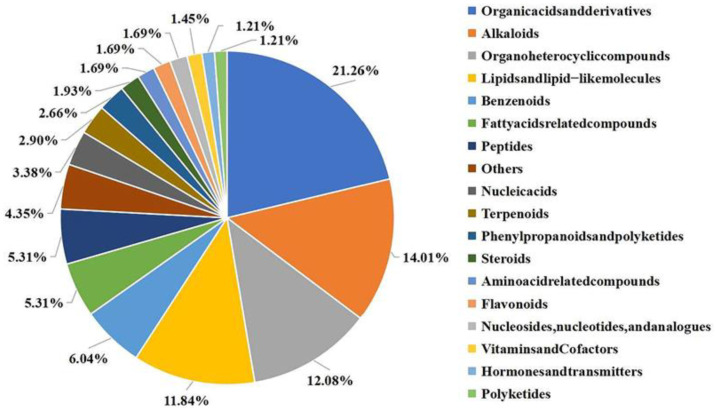
Relative proportions of different types of metabolites in J-15. Different colors represent different classifications of compounds and their proportions. All compounds with their affiliation to the particular group can be found in Table S5.

To investigate the components of J-15-Ms that regulate plant growth and determine their action mechanism, we examined the extracted J-15-Ms using LC-MS. We identified 414 metabolites, among which organic acids and their derivatives, organoheterocyclic compounds, lipids, lipid-like compounds, and alkaloids accounted for the highest proportion (more than 10% each). There were 27 benzenoids, 25 fatty acid-related compounds, 22 peptides, and 21 organic oxygen compounds (Figure 3). A few nucleic acids, terpenoids, organic nitrogen compounds, phenylpropanoids, flavonoids, amino acids, and nucleotides were also detected.

The metabolites produced by J-15 included several primary metabolites, such as organic acids, amino acids, nucleic acids, lipids, and carbohydrates, and secondary metabolites, such as alkaloids, flavonoids, phenylpropanoids, terpenoids, and phytohormones. These primary metabolites are indispensable for plant growth and development, while the secondary ones are closely related to plant disease, stress resistance, and growth regulation. Thus, these compounds synergistically regulate plant growth.

Alkaloids are a class of metabolites with complex ring structures and are mainly involved in preventing stress effects in plants. Up to 42 alkaloids were obtained in J-15-Ms and were presented in the order of their abundance, as shown in Table 1. 

Terpenoids are an important class of J-15-Ms consisting of isoprene as their basic component and can be used as phytoprobiotics to protect plants by reducing the infestation of pathogens and feeding animals and insects. The terpenoids identified in J-15-Ms included Gossypol, Glaucarubin, Withanolide, Agavoside A, Protodioscin, Musennin, and Soyasaponin A1 (Table 2). 

Flavonoids are a broad class of J-15-Ms with bacteriostatic, antioxidant, antitumor, immune-enhancing, and other biological activities. Flavonoids protect plants against UV light and regulate the transportation of plant growth hormones. Mulberrofuran A, Genistein, Prenyl glucoside, Daidzein, (R)-Glabridin, Glycitein, and Dihydrokaempferol were the identified J-15-Ms flavonoids (Table 3). 

Three plant hormones—indoleacetic acid (IAA), abscisic acid (ABA), and jasmine acid (JA)—and 14 indole plant growth regulators, such as indole-3-carboxaldehyde, indoleacetaldehyde, 3-Methylindole, indole-3-acetaldehyde, and 3-Methylindole, were detected in J-15-ms (Table 4).

**Figure 4:**
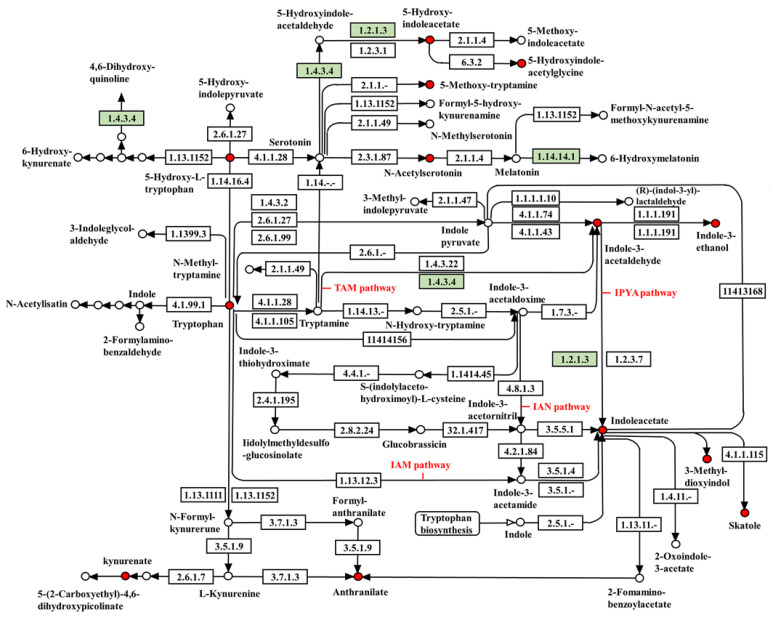
Diagram of the KEGG pathway of tryptophan metabolism from the J-15 genome combined with the metabolome. Red dots indicate that the material is the material tested in this LC-MS. Green markers are genes related to the IAA synthesis pathway identified in the J-15 genome. Not all target genes and metabolites are included in the figure.

The KEGG maps of the target metabolites involved in the IAA synthesis pathway and the analysis of the tryptophan metabolic pathway in the J-15 genome identified the possible involvement of J-15 in the IAA biosynthetic pathway (Figure 4). The results showed that genes involved in the IPyA and TAM pathways were present, while the key genes involved in the IAM and IAN pathways were lacking in the J-15 genome. Since tryptophan, indole-3-acetaldehyde, 3-indoleacetic acid, 3-methylindole, and indoleacetaldehyde, which are all J-15-Ms, play key roles in the IPyA and TAM pathways, it can be concluded that J-15-Ms can biosynthesize IAA via the IPyA and TAM pathways.

### 2.4. Effect of Mycosubtilin on Arabidopsis Growth

**Figure 5:**
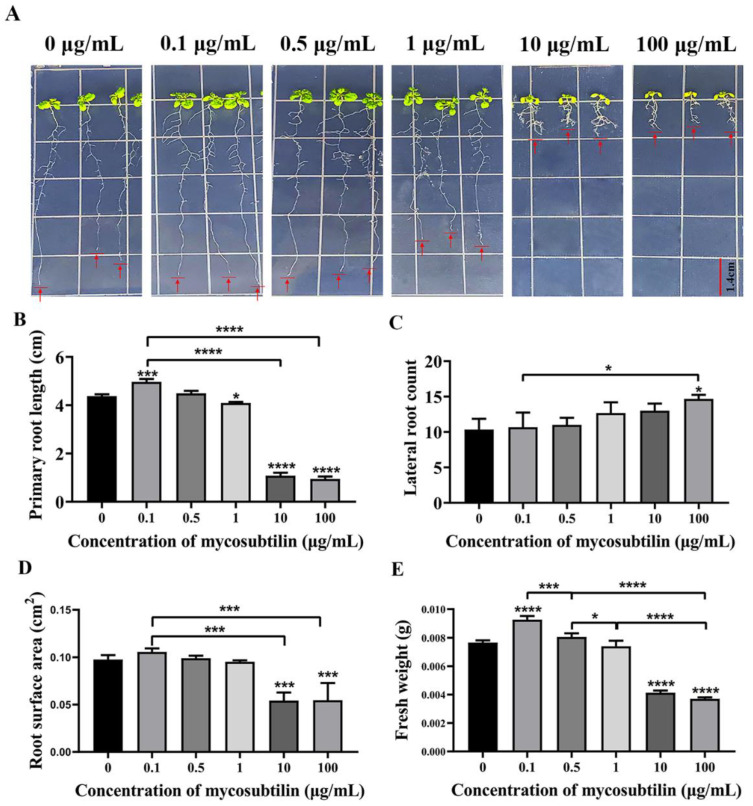
Effect of mycosubtilin on the growth of Arabidopsis seedlings. (**A**) Growth phenotype of Arabidopsis seedlings. (**B**) Effect on primary root length. (**C**) Effect on the number of lateral roots. (**D**) Effect on root surface area. (**E**) Effect on fresh weight. Note: Means ± SEs, n = 50, different “*” indicate significant differences among treatments based on the least significant difference at *p* ≤ 0.05.

*Arabidopsis* seedlings were treated with different concentrations of mycosubtilin for 10 days to investigate its effect on plant growth. The results showed that at a very low concentration of 0.1 μg/mL, mycosubtilin improved the growth of *Arabidopsis* seedlings, increasing their primary roots, root surface area, and fresh weight, compared to the control group. As the concentration increased (1 μg/mL), mycosubtilin inhibited the growth of the primary roots, the root surface area, and the fresh weight of *Arabidopsis* seedlings but enhanced the development of lateral roots (Figure 5). Thus, mycosubtilin also had the same low-concentration-promoting and high-concentration-inhibiting, but not lethal, effects on the growth and development of *Arabidopsis*.

### 2.5. Expression Pattern of the Genes Associated with Growth Regulatory Mechanisms of Mycosubtilin in Arabidopsis

**Figure 6:**
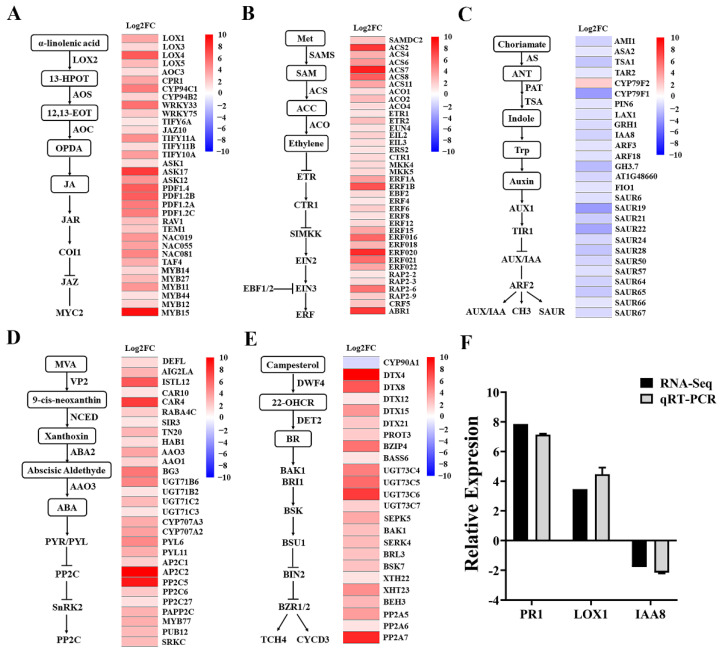
Fold changes in gene expression of major plant hormone signallingsignaling pathways by mycosubtilin treatment. After 12 h treatment with mycosubtilin in Arabidopsis: (**A**) JA signaling pathway fold change in gene expression; (**B**) ET signaling pathway fold change in gene expression; (**C**) IAA signaling pathway fold change in gene expression; (**D**) ABA signaling pathway fold change in gene expression; (**E**) BR signaling pathway fold change in gene expression; and (**F**) transcriptome data qRT-PCR verification.

Mycosubtilin exhibited significant inhibition of primary root growth and promotion of lateral root development in *Arabidopsis*. To investigate the plant growth regulatory mechanism by mycosubtilin, we treated *Arabidopsis* seedlings with 50 μg/mL of mycosubtilin for 10 days and analyzed the changes in gene expression of the phytohormone pathway (Figure 6). The results showed that the mycosubtilin treatment upregulated *LOX4*, *AOC3*, *CYP94C1, JAZ10*, *ASK17*, and *MYB15* genes of the JA signaling pathway in *Arabidopsis*. Similarly, *ACS2*, *ACS7*, *ETR2*, *ERS2*, *ERF18*, and *ERF20* of the ET signaling pathway and *DT4*, *DT8*, *UGT73*, and *PP2A* of the BR signaling pathway were highly upregulated. For the ABA signaling pathway, genes such as *CTR4*, *AAO3*, *BG3*, *PYL6*, and *AP2A* were upregulated. To ensure the reliability of the transcriptome data, *PR1*, *LOX1*, and *IAA8* of the phytohormone signaling pathway were verified by qRT-PCR, and the results showed consistent expression trends with the transcriptome data. Thus, mycosubtilin inhibited the primary root growth and promoted lateral root development in *Arabidopsis* by upregulating various genes involved in the JA, ET, BR, and ABA signaling pathways and downregulating those involved in the IAA signaling pathway. This suggests that mycosubtilin may affect plant growth by regulating phytohormone levels.

## 3. Discussion

PGPR is essential for improved plant growth, either through the direct secretion of plant growth and development-related substances or indirectly by regulating the living plant environment [45]. ACC deaminase-producing *Burkholderia cepacia* P10 promotes plant growth by producing compounds such as IAA and iron carriers [46]. Two PGPR strains from the pokeweed root zone, WM13-24 and M30-35, promote ryegrass growth and root development by regulating phytohormone distribution [47]. Moreover, *Bacillus pallidus* PP7S promotes plant growth and increases anthocyanin biosynthesis in *Arabidopsis* by triggering specific induced systemic resistance (ISR) in the plant [48]. Previous studies have shown that J-15-Ms can induce *V. dahliae* resistance in cotton [40] and inhibit various pathogenic bacteria [41]. In this study, *Arabidopsis* seedlings were treated with different concentrations of J-15-Ms for 10 days. The results showed that, at 0.2 μg/mL, J-15-Ms had a significant pro-growth effect on *Arabidopsis*, increasing the primary root length, lateral root number, root surface area, and fresh weight of the *Arabidopsis* seedlings compared to the control. However, as the concentration increased, the root surface area, root length, and lateral root number decreased, inhibiting the growth of *Arabidopsis* seedlings (Figure 1). This indicates that lower J-15-Ms concentrations promote the growth of *Arabidopsis* roots, while higher concentrations inhibit it. Similar results were also obtained on cotton growth analysis. The treatment with 1 μg/mL of J-15-Ms increased the biomass of the treated cotton compared to the control, and 10 μg/mL resulted in taller cotton plants with longer roots and an increased root-to-crown ratio. However, at the J-15-Ms concentration of 100 μg/mL, the cotton growth was inhibited, resulting in reduced root length, plant height, root-to-crown ratio, and biomass of the treated cotton compared to the control, and it even appeared that the roots turned black and the primary roots broke off (Figure 2). Although low concentrations of J-15-Ms are beneficial to the growth of cotton, too high concentrations can cause damage to the cotton, but this damage does not lead to death. This suggests that the J-15-Ms have regulatory effects on plant growth and development. Thus, we speculate that a certain component of J-15-Ms could be directly involved in plant growth and development promotion by regulating the dynamic balance of plant hormone levels and nutrients in the plant root system.

To investigate how the J-15-Ms regulate plant growth, we analyzed and identified the J-15-Ms components by LC-MS. The results showed that various organic acids and their derivatives, organic heterocyclic compounds, lipids, and lipid-like compounds, which are all primary metabolites related to plant growth and development, were contained among the J-15-Ms. There were also secondary metabolites, such as alkaloids, flavonoids, peptides, a few nucleic acids, terpenoids, phenylpropanoids, flavonoids, amino acids, and phytohormones (Figure 3). We found that the J-15-Ms exhibited broad-spectrum resistance to various pathogens [41]. The alkaloids xanthine, angularine, vincristine, caffeine, and 6-acetylmorphine found in the J-15-Ms (Table 1) also have excellent inhibitory effects against various plant pathogens [49,50,51]. The terpenoid (Table 2) gossypol can effectively inhibit the germination of *V. dahliae* spores and the growth of *V. striatum* [52]. Furthermore, saponins, such as agave saponin, proto-diosgenin, and soy saponin, have antibacterial, antiviral, and insecticidal effects and strong inhibitory activity against phytopathogenic bacteria causing wheat root rot [53,54]. The flavonoid (Table 3) photoglycyrrhizin also has inhibitory effects against various plant pathogens [55,56]. Genistein regulates plant growth and mutual recognition between plants and microorganisms, (R)-Glabridin inhibits various plant pathogens, and Glycitein exhibits concentration-dependent inhibition of plant radicle length. These substances indirectly promote plant growth by inhibiting the growth of pathogenic bacteria and inducing resistance in plants [57].

It has been reported that PGPR directly promotes plant growth by secreting growth-regulating substances such as IAA. *Pseudomonas aeruginosa* HMR1 produces IAA and, thus, has a high plant growth-promoting potential [58]. It was found that *Bacillus cereus* CJCL2 and RJGP41 could positively regulate the expression of plant hormones such as ABA, thereby significantly improving plant growth under cold stress [59]. Moreover, inoculation with *Pseudomonas* spp. significantly increased the levels of hormones such as ABA but reduced ET levels in soybean plants to enhance the drought stress tolerance of soybeans [60]. In this study, LC-MS analysis showed that J-15-SMs contain phytohormones, such as IAA, ABA, and JA, and that indole-3-acetaldehyde, 3-indoleacetic acid, 3-methylindole, and other plant growth regulators regulate plant growth (Table 4). IAA has a crucial regulatory role in almost all plant growth and development stages, especially root development and structure. At lower concentrations, IAA stimulates primary root elongation, but reduces primary root length, increases root hair formation, and stimulates the formation of lateral roots at higher concentrations. Overall, IAA regulates the surface area and length of roots, allowing plants greater access to soil nutrients [61]. Conversely, ABA inhibits seed germination and primary root growth by suppressing lateral root germination and the activity of lateral root meristems. ABA also inhibits lateral root growth and development by cross-interacting with various plant hormones and environmental signals [62]. Although it is an important hormone that enhances plant defense, higher levels of JA can inhibit plant growth. Indoles, such as indole-3-butyric acid, are also important broad-spectrum plant growth regulators. Since most indoles are intermediates in the synthesis of indoleacetic acid from tryptophan, J-15 could likely use tryptophan to synthesize IAA to regulate plant growth. When the IAA concentration is in the right range, it can stimulate the production of root hairs and increase the length and number of lateral roots in plants; however, the development of primary roots is inhibited to some extent when the concentration of produced IAA is high [63]. This is consistent with our previous results that low concentrations of J-15-Ms promote plant growth and high concentrations of J-15-Ms inhibit growth. Therefore, we considered that the key substance in J-15-Ms that regulates plant growth is IAA. The tryptophan-dependent IAA production is widely considered the most dominant mode of IAA secretion by plant bacteria [64]. Therefore, we performed a combined KEGG analysis of the J-15 genome and J-15-MS. The results showed that J-15 contained genes related to the IPyA and TAM pathway of tryptophan metabolism, and that tryptophan, indole-3-acetaldehyde, 3-indoleacetic acid, 3-methylindole, and indole acetaldehyde in the J-15-Ms were all present in the IAA synthesis pathway (Figure 4). This suggests that J-15 can use these substances to synthesize IAA. Thus, J-15 may regulate plant growth by promoting IAA biosynthesis through the IPyA and TAM pathways.

Mycosubtilin, a lipopeptide purified from J-15-Ms, has strong antifungal activity [44]; however, its effect on plant growth has not been reported. The treatment of *Arabidopsis* seedlings and cotton with different mycosubtilin concentrations showed the same pattern of pro-growth at lower concentrations and inhibition at higher concentrations. At a lower concentration of 0.1 μg/mL, the growth of *Arabidopsis* seedlings was promoted, increasing the primary root length, root surface area, and fresh weight compared to the control. However, the plant growth decreased as the concentration increased; for example, mycosubtilin treatment at 1 μg/mL inhibited the primary root length, root surface area, and fresh weight but promoted the development of lateral roots of the *Arabidopsis* seedlings. Thus, the higher the concentration, the more pronounced the growth inhibition was. Although higher mycosubtilin concentrations strongly inhibited *Arabidopsis* growth, it did not cause lethal effects at 100 μg/mL (Figure 5). 

To elucidate the molecular mechanism of the growth-regulating effect of mycosubtilin, we analyzed the genes regulating plant growth using the *Arabidopsis* transcriptome data obtained after 12 h of mycosubtilin treatment. We found that mycosubtilin treatment upregulated the expression of genes in the JA, ET, ABA, and BR signaling pathways and downregulated the expression of those in the IAA signaling pathway. In the JA signaling pathway, the expression of *LOX1*, *LOX4*, *AOC3*, *CYP94*, and other genes regulating jasmonic acid synthesis were upregulated. When synthesized in large quantities, JA significantly inhibits the elongation of *Arabidopsis* primary roots in a concentration-dependent manner, with higher concentrations causing severe inhibition of root growth [65]. JA regulates the growth of lateral roots via the JA-COI1-JAZ pathway, and the JAZs proteins downstream of JA form a complex with transcription factors, such as RHD6 and RSL1, which regulate root hair development [66]. Mycosubtilin treatment significantly upregulated *JAZ10*, *ASK17*, *PDF1.2*, and *MYB15* genes of the JA-COI1-JAZ pathway, which inhibited primary root growth and promoted lateral root development. ET promotes the biosynthesis and transport of growth hormone, leading to local accumulation of growth hormone in the plant, thus inhibiting cell elongation and root growth [67]. Several ET receptor genes, such as *ETR1*, *ETR2*, and *ERS2*, were upregulated by mycosubtilin treatment, activating the ET response [68]. Similarly, several genes of the ERF family of ethylene response factors were upregulated by mycosubtilin, promoting the regulatory effect of ET on the plant root system. IAA plays a crucial role in plant development, such as embryo development, root development, apical dominance, and various directional responses, which can be regulated through the IAA signaling pathway [69]. *PIN6*, *IAA8*, *ARF3*, and *ARF18* genes are the key regulatory components exerting multiple IAA effects on plant growth and development [70]. The IAAs and ARFs interacting with AUX/IAAs play an important role in lateral root development [71,72]. Furthermore, downregulating GH3 and SUAR family genes downstream of IAA inhibits the growth of primary roots and promotes the development of lateral and adventitious roots by regulating the transportation of growth hormones [73,74]. ABA is involved in many plant growth and development processes, such as the inhibition of plant seed germination and primary root growth. Mycosubtilin treatment upregulated the expression of *CAR4* and *AAO3* genes of the ABA pathway, which promoted ABA synthesis [75]. The ABA-regulated lateral root growth in *Arabidopsis* requires the activation of ABA receptor PYL family genes [76], and *MYB77* can be activated by the growth hormone to promote lateral root growth. Furthermore, interactions between *PYL8* (of the PYL family) and *MYB77* regulate lateral root growth recurrence after inhibition [77]. BR is essential for normal plant growth and development [78]. Studies have shown that BR plays an important regulatory role in maintaining the size of *Arabidopsis* root meristems, root cell elongation, lateral root primordia initiation, root hair formation, and root weight orientation [79]. In the presented study, mycosubtilin treatment upregulated the expression of *DTX4*, *DTX8*, *PROT3*, and other genes that promote BR production. Upregulation of *BAK1*, *BRL3*, and *BSK7* genes of the BR signaling pathway, such as *BRI1* and *BSKs*, has been shown to promote endogenous BR levels and root elongation [80]. As part of the BR signaling pathway, the BRI1-related receptor kinase *BAK1* is involved in root development, and its mutants show a root-inhibiting phenotype [81]. Moreover, BR, IAA, ET, and other hormonal signals can synergistically or antagonistically regulate the growth and development of *Arabidopsis* roots [82]. In summary, the mycosubtilin treatment activated gene expression changes in the IAA, JA, ET, ABA, and BR signaling pathways, and these hormonal pathways acted synergistically to inhibit primary root and promote lateral root development in *Arabidopsis*.

## 4. Materials and Methods

### 4.1. Strain Activation and Extraction of Metabolites

*Bacillus subtilis* J-15 was isolated from the rhizosphere soil of a healthy continuous cotton field in Xinjiang and stored in the key laboratory of special species conservation and regulatory biology at Xinjiang Normal University, China. *Bacillus subtilis* J-15 was activated by scribing, and single colonies were picked and cultured in beef paste peptone medium at 37 °C with shaking at 180 revolutions/min for 18 h. The J-15-Ms were extracted with acetone, chloroform (to remove the heteroproteins), and n-butanol [83]. The J-15-Ms was freeze-dried and solubilized using dimethyl sulfoxide, then purified by high-performance liquid chromatography to obtain the lipopeptide mycosubtilin [44].

### 4.2. Cultivation and Treatment of Arabidopsis Seedlings

This study used the wild-type *Arabidopsis*, Columbia-0 (Col-0), obtained from the key laboratory of special species conservation and regulatory biology at Xinjiang Normal University, China. The 7-day *Arabidopsis* Col-0 seedlings grown on MS (Murashige and Skoog) medium were transferred to a new Petri dish with 1/2 MS medium and grown in a controlled light incubator (GXM-508, produced in Ningbo Jiangnan Instrument Factory, China) at 23 ± 1 °C under 50 μmol/m^2^·s photon flux density of photosynthetically active radiation with a relative humidity of 70% under 18 h light/6 h dark photoperiod.

### 4.3. Cultivation and Treatment of Cotton Seedlings

The cotton strain Xinluzao 72 used in this study was kindly donated by the Xinjiang Academy of Agricultural Sciences, China. The full-grained lint-free cotton seeds were surface disinfected with 2% sodium hypochlorite for 20 min and rinsed 4–5 times in sterile water. The seeds were soaked in sterile water for 6–12 h and wrapped in moist triple gauze for germination. After the germination, the seeds were transferred in a sterile soil bowl (containing vermiculite: nutrient soil: flower soil = 1:1:2) and were incubated in a plant culture room at 25 °C/21 °C day/night temperature under 75 μmol/m^2^·s photon flux density of photosynthetically active radiation, relative humidity of 60–75%, and 16 h day/8 h dark photoperiod, with regular watering.

### 4.4. Growth Indicators in Arabidopsis Treated with J-15-Ms

Three-day-old *Arabidopsis* Col-0 seedlings, grown on MS medium, were transferred to Petri dishes containing 1/2 MS medium with different concentrations of J-15-Ms (0 μg/mL, 0. 2 μg/mL, 2 μg/mL, and 20 μg/mL), with three replicates for each concentration, containing at least 50 *Arabidopsis* seedlings in each concentration. The plates were kept vertically so that the roots of the plants grew downwards at 23 ± 1 °C under 50 μmol/m^2^·s photon flux density of photosynthetically active radiation with a relative humidity of 70% under 18 h light/6 h dark photoperiod. After 7 days, images of the *Arabidopsis* seedlings were taken, and their growth indicators, such as primary root length, lateral roots, and root surface area, were measured at different concentrations using ImageJ software. 

### 4.5. Growth Indicators in Arabidopsis Treated with J-15-Ms

The leaf surface of the cotton seedlings at the two-leaf and one-center stages were sprayed with different concentrations of J-15-Ms (0 μg/mL, 1 μg/mL, 10 μg/mL, and 100 μg/mL) once a day for three days under 75 μmol/m^2^·s photon flux density of photosynthetically active radiation, relative humidity of 60–75%, and 16 h day/8 h dark photoperiod, with regular watering. At least 20 cotton plants were in each experiment, and three replicates were performed. After 21 days of treatment, the growth indicators, such as root length, plant height, fresh weight, and dry weight, were measured according to the method used in Section 4.4.

### 4.6. Full-Spectrum LC-MS Detection of J-15-Ms

A mass of J-15-Ms was extracted at low temperature, accurately measured, and then centrifuged to extract the supernatant metabolite solvent for liquid–liquid mass spectrometry. The sample processing or chromatographic conditions (http://www.majorbio.com/, accessed on 21 September 2020). included: ACQUITY UPLC HSS T3 (100 mm × 2.1 mm i.d 1.8 µm; Waters, Milford, USA), mobile phase A, and mobile phase B (Table S1). The mobile phase A was 95% water + 5% acetonitrile containing 0.1% formic acid, while the mobile phase B was 47 5% acetonitrile + 47.5% isopropanol + 5% water containing 0.1% formic acid. The injection volume and the column temperature were 2 µL and 40 °C, respectively, and the mobile phase elution gradient is shown in Table S2. Mass spectrometric conditions: the samples were subjected to electrospray ionization, and the mass spectrometric signals were acquired in positive and negative ion scanning mode, respectively, with the parameters shown in Table S3.

### 4.7. Growth Indicators in Arabidopsis Treated with Mycosubtilin

Mycosubtilin concentrations were set at 0 μg/mL, 0.1 μg/mL, 0.5 μg/mL, 1 μg/mL, 5 μg/mL, 10 μg/mL, and 100 μg/mL, with three replicates for each concentration, containing at least 50 *Arabidopsis* seedlings in each concentration. Three-day-old *Arabidopsis* Col-0 seedlings grown on MS medium were transferred to different concentrations of mycosubtilin in Petri dishes, and the plates were kept vertically to allow for downwards growth of the roots. After 7 days, images of the seedlings were taken, and their growth indicators, such as primary root length, lateral root length, and root surface area, were measured as described in Section 4.4.

### 4.8. Gene Expression of the Hormone Signaling Pathways of Arabidopsis Treated with Mycosubtilin

*Arabidopsis* Col-0 seedlings grown on MS medium for 10 days were planted in six-well plates containing 50 μg/mL mycosubtilin, and those planted in plates with dd H_2_O served as controls. The whole plant samples were collected at 12 h after treatment and sent to Shanghai Meiji Biologicals (http://www.majorbio.com/, accessed on 31 July 2020), China, for transcriptome sequencing. Three biological replicates were performed per sample. 

*Arabidopsis* Col-0 seedlings were treated with 50 μg/mL mycosubtilin for 12 h. Total RNA was extracted from each group of samples by TRIzol regent (Tiangen, Beijing, China), and the nucleic acid concentration was measured using an ultra-micro spectrophotometer (Thermo Fisher™ NanoDrop One, Waltham, MA, USA). The RNA was reverse transcribed into cDNA using a reverse transcription kit (Tiangen, Beijing, China ), The qRT-PCR reaction was performed on the Real-Time PCR instrument (ABI StepOne™, FosterCity, CA; USA). Data were standardized using *AtActin* and three replicates were made. Specific primers were designed according to the gene sequence, and primer sequences are shown in Table S4.

### 4.9. Statistics

Data were subjected to one-way analysis of variance (ANOVA) using SPSS 20 (IBM, Armonk, NY, USA). The significant differences between treatments were tested according to the least significant difference (LSD) at *p* ≤ 0.05. The asterisk “*” indicates a significant difference between the groups.

## 5. Conclusions

This study found that lower J-15-Ms concentrations promoted the growth of *Arabidopsis* and cotton seedlings, while higher concentrations inhibited the growth. LC-MS analysis found that J-15 regulates plant growth by synthesizing IAA via the IPyA and TAM pathways. The mycosubtilin purified from J-15-Ms also promoted plant growth at lower concentrations and inhibited growth but promoted lateral root development at higher concentrations. The transcriptome data showed that mycosubtilin regulates plant growth by upregulating the genes associated with JA, ET, ABA, and BR pathways and downregulating IAA pathway-related genes. Therefore, these findings provide preliminary insights into the growth-promoting mechanism of J-15 and a scientific basis for developing efficient.

## Figures and Tables

**Table 1:** Alkaloids in J-15-Ms.

Metabolite	*m*/*z*	Mode	Formula	Peak Area
Xanthine	151.0249	neg	C_5_H_4_N_4_O_2_	465,374.5
Angularine	352.1755	pos	C_18_H_25_NO_6_	454,052.7
Ecgonine methyl ester	241.1547	pos	C_10_H_17_NO_3_	417,993.9
Fagomine	192.0618	pos	C_6_H_13_NO_3_	345,944.4
Vincristine	861.3502	neg	C_46_H_56_N_4_O_10_	238,339
Caffeine	195.0876	pos	C_8_H_10_N_4_O_2_	200,524.5
Cardiopetalidine	364.2455	pos	C_21_H_33_NO_4_	195,000.2
Phenyllactic acid	165.0545	neg	C_9_H_10_O_3_	185,594.5
Nicotyrine	159.0916	pos	C_10_H_10_N_2_	126,128.4
Europine	330.1915	pos	C_16_H_27_NO_6_	106,925.8
6-Acetylmorphine	699.2957	neg	C_19_H_21_NO_4_	102,800.9
Petasitenine	364.1755	pos	C_19_H_27_NO_7_	917,93.65
Monocrotaline	326.1601	pos	C_16_H_23_NO_6_	69,254.51
Otonecine	186.1125	pos	C_9_H_15_NO_3_	68,611.49
Hypoxanthine	137.0457	pos	C_5_H_4_N_4_O	60,381.4

**Table 2:** Terpenoids in J-15-Ms.

Metabolite	*m*/*z*	Mode	Formula	Peak Area
Gossypol	519.2048	pos	C_30_H_30_O_8_	649,034
Glaucarubin	495.2212	neg	C_25_H_36_O_10_	112,114.8
Withanolide	484.2761	pos	C_28_H_38_O_6_	119,294.7
Agavoside A	627.3294	neg	C_33_H_52_O_9_	233,633.7
Protodioscin	1029.526	neg	C_51_H_84_O_22_	3,087,680
Musennin	1031.541	pos	C_51_H_82_O_21_	2,271,846
Soyasaponin A1	633.2946	neg	C_59_H_96_O_29_	70,810.22
Soyasaponin I	941.5121	neg	C_48_H_78_O_18_	2,321,121
(2R,6S,7S,10Z)-beta-Santala-3(15),10-dien-12-ol	284.1968	pos	C_15_H_24_O	33,043.89
(+)-(S)-Carvone	195.1016	neg	C_10_H_14_O	29,834.57
Convalloside	735.3313	pos	C_35_H_52_O_15_	4,774,323

**Table 3:** Flavonoids in J-15-Ms.

Metabolite	*m*/*z*	Mode	Formula	Peak Area
Mulberrofuran A	434.2291	pos	C_25_H_28_O_4_	32,728.46
Genistein	271.06	pos	C_15_H_10_O_5_	13,176.96
Prenyl glucoside	293.1241	neg	C_11_H_20_O_6_	120,163.7
Daidzein	255.0652	pos	C_15_H_10_O_4_	67,927.36
(R)-Glabridin	647.2687	neg	C_20_H_20_O_4_	76,256.28
Glycitein	285.0757	pos	C_16_H_12_O_5_	28,548.9
Dihydrokaempferol	269.0456	neg	C_15_H_12_O_6_	13,101.75

**Table 4:** Hormonal substance in J-15-Ms.

Metabolite	*m*/*z*	Mode	Formula	Peak Area
Indole	118.0653	pos	C_8_H_7_N	540,110.20
3-Methyloxindole	148.0756	pos	C_9_H_9_NO	192,913.81
3-Methyleneoxindole	146.0599	pos	C_9_H_7_NO	115,995.00
Indole-3-carboxaldehyde	146.0599	pos	C_9_H_7_NO	96,755.36
Indoleacetaldehyde	160.0756	pos	C_10_H_9_NO	83,635.57
5-Hydroxyindoleacetylglycine	213.0659	pos	C_12_H_12_N_2_O_4_	45,844.55
3-Methylindole	176.0706	neg	C_9_H_9_N	37,102.86
1H-Indole-3-carboxaldehyde	144.0443	neg	C_9_H_7_NO	31,995.86
Indole-3-carboxylic acid	144.0444	pos	C_9_H_7_NO_2_	31,241.96
Indolelactic acid	204.0659	neg	C_11_H_11_NO_3_	25,155.98
3-Methyldioxyindole	144.0441	neg	C_9_H_9_NO_2_	23,865.93
3-Indoleacetic Acid	174.055	neg	C_10_H_9_NO_2_	14,909.82
5-Hydroxyindoleacetic acid	190.0501	neg	C_10_H_9_NO_3_	14,043.72
Abscisic acid	229.1221	pos	C_15_H_20_O_4_	10,233.48
Indole-3-acetaldehyde	201.1021	pos	C_10_H_9_NO	9261.27
5,6-Dihydroxyindole	194.0464	neg	C_8_H_7_NO_2_	2551.33
Jasmonic acid	191.1068	neg	C_12_H_18_O_3_	2035.92

## Data Availability

The datasets supporting the results of this article are available in the CGNB Sequence Archive (CNSA) of China National GeneBank DataBase (CNGBdb) with accession number: CNP0003287. (https://db.cngb.org/search/project/CNP0003287/ (accessed on 27 August 2022)).

## Supplementary Materials

The following supporting information can be downloaded at: https://www.mdpi.com/article/10.3390/plants11233205/s1, Table S1: Main reagents; Table S2. Mobile phase elution gradients; Table S3. Mass spectrometry parameters; Table S4. qRT-PCR primer sequences (Sangon Biotech); Table S5 Classification of compounds identified by LC-MS.
