# Supplementary material for: Metabolites from Bacillus subtilis J-15 Affect Seedling Growth of Arabidopsis thaliana and Cotton Plants
Source: Plants (Basel). 2022 Nov 23;11(23):3205. doi: 10.3390/plants11233205 (PMC9739671; doi:10.3390/plants11233205)
Supplement: Supplementary file 1 [file plants-11-03205-s001.zip › plants-2023399-supplementary.pdf]

Supplementary Materials

Table S1. Main reagents

Reagents and supplies	Level	CAS	Manufacturers
Methanol	HPLC	67-56-1	Fisher Chemical
Acetonitrile	HPLC	75-05-8	Fisher Chemical
Formic Acid	HPLC	64-18-6	CNW
Water	LC-MS	7732-18-5	Fisher Chemical
2-Propanol	HPLC	67-63-0	Merck
2-Chloro-L-Phenylalanine	≥98%	103616-89-3	Adamas-beta

Table S2. Mobile phase elution gradients

Time（min）	Flow rate（mL/min）	A （%）	B （%）
0	3	0	100
0	3	60	40
40	3	50	50
50	3	0	100
60	3	0	100

Table S3. Mass spectrometry parameters

Description	Parameter
Scan type（m/z）	70-1050
Sheath gas flow rate（arb）	50
Aux gas flow rate（arb）	13
Heater temp（℃）	425
Capillary temp（℃）	325
Spray voltage（+）（V）	3500
Spray voltage（-）（V）	-3500
S-Lens RF Level	50
Normalized collision energy（eV）	20,40,60
Resolution（Full MS）	60000
Resolution（MS^2）	7500

Table S4. qRT-PCR primer sequences (Sangon Biotech)

Target gene	primer (5'→3')
*At*ACTF	GCCGACAGAATGAGCAAAGAG

*At*ACTR	AGGTACTGAGGGAGGCCAAGA
*At*PR1F	ACGGGGAAAACTTAGCCTGG
*At*PR1R	TTGGCACATCCGAGTCTCAC
*At*LOX1F	GTGGATGGGCTTGAGGTTTGGTAT
*At*LOX1R	TTCTTCACGGGTTTGCATTTTAGG
*At*IAA8F	ATGCTATCGCCTAGACCTGTT
*At*IAA8R	TGCCTTAGATGCTGGCTGTG

Table S5 Classification of compounds identified by LC-MS

Compound	Metabolite	class
1.09_137.0457m/z	Hypoxanthine	Alkaloids
0.90_151.0249m/z	Xanthine	Alkaloids
5.05_300.1918m/z	DL-Pipecolinic acid	Alkaloids
1.52_259.1652m/z	L-Pipecolic acid	Alkaloids
4.59_220.0610m/z	Indoleacetic acid	Alkaloids
3.93_174.0550m/z	3-Indoleacetic Acid	Alkaloids
3.21_184.0255m/z	6-Hydroxynicotinic acid	Alkaloids
3.98_166.0617n	Phenyllactic acid	Alkaloids
1.33_283.0682m/z	Xanthosine	Alkaloids
2.31_148.1120m/z	Pseudoephedrine	Alkaloids
2.98_183.0918m/z	Anabasine	Alkaloids
1.00_142.1225m/z	PELLETIERINE	Alkaloids
4.61_200.1284m/z	Methylisopelletierine	Alkaloids
2.55_214.1103n	Harmaline	Alkaloids
7.85_861.3502m/z	Vincristine	Alkaloids
0.60_194.0803n	Caffeine	Alkaloids
2.30_140.1069m/z	(S)-Homostachydrine	Alkaloids
6.29_364.2455m/z	Cardiopetalidine	Alkaloids
4.03_356.1858m/z	Glaucine	Alkaloids
3.34_154.0862m/z	Arecoline	Alkaloids
6.91_443.3630m/z	Carpaine	Alkaloids
5.74_202.1230m/z	Dioscorine	Alkaloids
8.36_192.0618m/z	Fagomine	Alkaloids
2.95_179.0946n	Fusaric acid	Alkaloids
3.52_159.0916m/z	Nicotyrine	Alkaloids
5.97_285.1815m/z	Proline betaine	Alkaloids
4.93_284.1856m/z	Amabiline	Alkaloids

4.24_352.1755m/z	Angularine	Alkaloids
2.74_330.1915m/z	Europine	Alkaloids
3.92_310.1652m/z	Fulvine	Alkaloids
3.38_326.1601m/z	MONOCROTALINE	Alkaloids
2.28_185.1051n	Otonecine	Alkaloids
3.27_364.1755m/z	Petasitenine	Alkaloids
3.16_307.1077m/z	Furofoline	Alkaloids
2.63_335.1359m/z	Ribalinium	Alkaloids
4.96_328.0938m/z	Rutacridone	Alkaloids
5.24_256.1543m/z	Meteloidine	Alkaloids
1.17_241.1547m/z	Ecgonine methyl ester	Alkaloids
3.82_184.0970m/z	Pseudoecgonine	Alkaloids
5.85_342.1699m/z	6-Monoacetylmorphine	Alkaloids and derivatives
1.11_699.2957m/z	6-Acetylmorphine	Alkaloids and derivatives
6.32_370.1084m/z	Xi-8-Acetonyldihydrosanguinarine	Alkaloids and derivatives
0.51_114.1028m/z	N-Carbamoylputrescine	Amino acid related compounds
0.74_444.0228m/z	Glucotropaeolin	Amino acid related compounds
2.09_531.2776m/z	Hordatine A	Amino acid related compounds
3.93_423.1745n	Neolinustatin	Amino acid related compounds
2.82_450.0708m/z	Glucocleomin	Amino acid related compounds
3.04_347.1237m/z	Miraxanthin-V	Amino acid related compounds
3.74_361.1395m/z	3-Methoxytyramine-betaxanthin	Amino acid related compounds
4.08_231.0770m/z	Nalidixic acid	Antibiotics
5.13_155.0351m/z	Catechol	Benzenoids
4.16_120.0445m/z	2-Aminobenzoic acid	Benzenoids
1.83_138.0549m/z	Anthranilic acid	Benzenoids
3.49_95.0496m/z	Phenol	Benzenoids
1.65_106.0422n	Benzaldehyde	Benzenoids
0.68_94.0656m/z	Aniline	Benzenoids
2.62_121.0649m/z	Phenylacetaldehyde	Benzenoids
3.30_153.0182m/z	Gentisic acid	Benzenoids
2.60_109.0650m/z	Anisole	Benzenoids
3.01_93.0703m/z	Toluene	Benzenoids
4.19_109.0650m/z	P-cresol	Benzenoids

4.32_162.0549m/z	Hippurate	Benzenoids
3.28_165.0182m/z	Phthalic acid	Benzenoids
3.16_124.0527n	3-Hydroxybenzyl alcohol	Benzenoids
2.40_198.1124m/z	Metanephrine	Benzenoids
2.73_137.0597m/z	Benzylformate	Benzenoids
1.04_120.0576n	P-Tolualdehyde	Benzenoids
3.16_105.0702m/z	Styrene	Benzenoids
0.71_212.1281m/z	Metaproterenol	Benzenoids
1.67_150.1277m/z	Methamphetamine	Benzenoids
1.18_237.0869m/z	O-Hydroxyhippuric acid	Benzenoids
6.19_257.1753m/z	Tetradecanedioic acid	Benzenoids
6.19_170.0962m/z	Diphenylamine	Benzenoids
4.57_207.1016m/z	3-Dimethylallyl-4-hydroxybenzoate	Benzenoids
5.24_259.1441m/z	4-Nonylphenol	Benzenoids
0.62_342.1152n	Sucrose	Carbohydrates
2.29_299.1372n	Saxitoxin	Cyanotoxins
6.26_193.0989m/z	Pelargonic acid	FA Fatty acyls
6.49_200.2006m/z	Decyl alcohol	FA Fatty acyls
6.45_228.2318m/z	Dodecanol	FA Fatty acyls
5.88_326.1727m/z	L-Octanoylcarnitine	FA Fatty acyls
3.31_218.1386m/z	O-propanoyl-carnitine	FA Fatty acyls
2.30_455.2382n	20-Hydroxy-leukotriene E4	FA Fatty acyls
2.18_352.2224n	20-Hydroxy-leukotriene B4	FA Fatty acyls
5.14_188.1039n	Azelaic acid	FA Fatty acyls
6.27_215.1643m/z	12-Hydroxydodecanoic acid	FA Fatty acyls
4.22_518.1994m/z	N-Acetyl-leukotriene E4	FA Fatty acyls
2.76_172.0968m/z	N-butanoyl-lhomoserine lactone	FA Fatty acyls
4.40_216.1230m/z	2-amino-8-oxo-9,10-epoxy-decanoic acid	FA Fatty acyls
7.04_283.2873n	Stearamide	FA Fatty acyls
6.76_283.2870n	Octadecanamide	FA Fatty acyls
3.77_316.2024n	15-Deoxy-d-12,14-PGJ2	FA Fatty acyls
5.95_331.1661m/z	13-OxoODE	FA Fatty acyls
6.11_330.2402n	9,12,13-TriHOME	FA Fatty acyls
6.29_315.2535m/z	(9S,10S)-9,10-dihydroxyoctadecanoate	FA Fatty acyls
6.06_316.3206m/z	Nonadecanoic acid	FA Fatty acyls
6.20_287.2224m/z	Pentadecanoic acid	FA Fatty acyls
7.90_565.5190m/z	Vaccenic acid	Fatty acids related compounds
4.75_191.1068m/z	Jasmonic acid	Fatty acids related compounds
5.91_269.0456m/z	Dihydrokaempferol	Flavonoids
4.52_434.2291m/z	Mulberrofuran A	Flavonoids
5.91_271.0600m/z	Genistein	Flavonoids
4.06_293.1241m/z	Prenyl glucoside	Flavonoids

5.50_255.0652m/z	Daidzein	Flavonoids
4.77_647.2687m/z	(R)-Glabridin	Flavonoids
5.49_285.0757m/z	Glycitein	Flavonoids
2.91_196.0971m/z	Tenuazonic acid	Fungal toxins
6.01_888.5183m/z	Paxilline	Fungal toxins
6.07_338.1957m/z	Alpha-Zearalenol	Fungal toxins
4.73_746.4447m/z	Fasciculol E	Fungal toxins
4.67_649.3560m/z	Janthitrem B	Fungal toxins
6.87_493.3498m/z	TG(8:0/8:0/8:0)	GL Glycerolipids
6.13_97.9773n	Phosphoric acid	Homogeneous non-metal compounds
0.66_370.1726m/z	Coutaric acid	Homogeneous non-metal compounds
0.63_146.1174m/z	Acetylcholine	Hormones and transmitters
3.12_362.1678n	Thyrotropin releasing hormone	Hormones and transmitters
6.25_350.2684m/z	17a-Hydroxypregnenolone	Hormones and transmitters
5.77_621.3031m/z	Isotetrandrine	Lignans, neolignans and related compounds
6.38_343.1211m/z	Enterolactone	Lignans, neolignans and related compounds
7.40_764.5160m/z	PC(15:0/18:2(9Z,12Z))	Lipids
7.42_758.5680m/z	PC(14:0/20:2(11Z,14Z))	Lipids
7.68_760.5840m/z	PC(14:0/20:1(11Z))	Lipids
6.33_301.2379m/z	Palmitic acid	Lipids
7.69_671.4600m/z	PA(16:0/16:0)	Lipids
8.07_719.4871m/z	PA(16:0/18:1(11Z))	Lipids
7.54_716.5442n	SM(d18:0/16:1(9Z)(OH))	Lipids
6.51_370.2578m/z	Prostaglandin E2	Lipids
6.75_326.3047m/z	Stearic acid	Lipids
2.17_625.3064n	Leukotriene C4	Lipids
6.45_522.3555m/z	LPC(18:1)	Lipids
1.70_306.6528m/z	LysoPC(22:6(4Z,7Z,10Z,13Z,16Z,19Z))	Lipids
1.51_584.3133m/z	LysoPC(20:2(11Z,14Z))	Lipids
5.89_570.3504m/z	LysoPC(20:1(11Z))	Lipids
6.54_508.3754m/z	LysoPC(P-18:0)	Lipids
2.29_541.2627m/z	Leukotriene D4	Lipids
3.07_474.2091m/z	Leukotriene E4	Lipids
6.11_1001.6293m/z	Prostaglandin J2	Lipids
6.19_413.2177m/z	11-Dehydro-thromboxane B2	Lipids

7.53_790.4581m/z	3-O-Sulfogalactosylceramide (d18:1/14:0)	Lipids
5.16_273.1808m/z	Myristic acid	Lipids
6.25_249.1126m/z	Ubiquinone-1	Lipids and lipid-like molecules
6.36_285.2430m/z	Palmitaldehyde	Lipids and lipid-like molecules
1.17_213.1237m/z	D-Dethiobiotin	Lipids and lipid-like molecules
2.75_175.0602m/z	2-Isopropylmalic acid	Lipids and lipid-like molecules
2.16_231.0868m/z	Glutarate semialdehyde	Lipids and lipid-like molecules
6.39_370.2584m/z	(13E)-11a-Hydroxy-9,15-dioxoprost-13-enoic acid	Lipids and lipid-like molecules
7.39_547.4121m/z	2-Hexaprenyl-6-methoxy-1,4-benzoquinone	Lipids and lipid-like molecules
6.08_434.2102n	Flunisolide	Lipids and lipid-like molecules
6.29_324.2529m/z	Oxandrolone	Lipids and lipid-like molecules
2.82_372.1879m/z	Prednisone	Lipids and lipid-like molecules
6.60_578.4153m/z	Hyperforin	Lipids and lipid-like molecules
6.09_286.2735m/z	Cyclohexaneundecanoic acid	Lipids and lipid-like molecules
6.16_414.2038n	Eplerenone	Lipids and lipid-like molecules
4.81_374.1709m/z	Trilostane	Lipids and lipid-like molecules
4.55_507.2157m/z	Difluprednate	Lipids and lipid-like molecules
6.66_334.2478n	Tetrahydrodeoxycorticosterone	Lipids and lipid-like molecules
0.78_202.1801m/z	Undecylenic acid	Lipids and lipid-like molecules
6.30_258.1827n	Diisobutyl adipate	Lipids and lipid-like molecules
6.05_478.3169m/z	Lithocholic acid glycine conjugate	Lipids and lipid-like molecules
6.08_434.2103n	DEXAMETHASONE ACETATE	Lipids and lipid-like molecules
5.97_393.2069m/z	Dexamethasone	Lipids and lipid-like molecules

6.49_1016.6243m/z	Tauroursodeoxycholic acid	Lipids and lipid-like molecules
2.25_230.1287n	Furanodienone	Lipids and lipid-like molecules
5.35_271.1077m/z	Furanogermenone	Lipids and lipid-like molecules
7.48_383.3525m/z	Cerebronic acid	Lipids and lipid-like molecules
6.96_282.2788m/z	Oleamide	Lipids and lipid-like molecules
5.25_315.1715m/z	12S-HHT	Lipids and lipid-like molecules
3.24_432.7507m/z	Azaspiracid	Marine biotoxins
7.38_903.4552m/z	Pectenotoxin 2	Marine biotoxins
0.76_347.0626n	Adenosine Monophosphate	Nucleic acids
0.74_305.0175m/z	Uridine 5'-monophosphate	Nucleic acids
0.78_113.0348m/z	Uracil	Nucleic acids
0.68_136.0618m/z	Adenine	Nucleic acids
1.18_126.0431n	Thymine	Nucleic acids
0.67_243.0976m/z	Thymidine	Nucleic acids
0.64_306.0491m/z	DCMP	Nucleic acids
0.68_152.0566m/z	Guanine	Nucleic acids
0.94_244.0690n	Uridine	Nucleic acids
0.73_330.0605m/z	Deoxyadenosine monophosphate	Nucleic acids
1.05_321.0489m/z	DTMP	Nucleic acids
0.69_112.0508m/z	Cytosine	Nucleic acids
1.07_284.0989m/z	Guanosine	Nucleic acids
1.70_246.1084m/z	Deoxyuridine	Nucleic acids
1.09_267.0732m/z	Inosine	Nucleosides, nucleotides, and analogues
1.18_146.0599m/z	Indole-3-carboxaldehyde	Nucleosides, nucleotides, and analogues
0.75_348.0703m/z	Adenosine 3'-monophosphate	Nucleosides, nucleotides, and analogues
2.47_222.0878m/z	5-Methyldeoxycytidine	Nucleosides, nucleotides, and analogues
3.10_438.0460m/z	6-Methylthioguanosine monophosphate	Nucleosides, nucleotides, and analogues

2.37_295.0937m/z	5-Fluorouridine	Nucleosides, nucleotides, and analogues
2.56_278.1134m/z	5'-Deoxy-5-fluorocytidine	Nucleosides, nucleotides, and analogues
0.79_205.0344m/z	Oxoadipic acid	Organic acids
1.67_159.0651m/z	Pimelic acid	Organic acids
4.04_173.0809m/z	Suberic acid	Organic acids
0.62_131.0697n	Creatine	Organic acids and derivatives
2.12_141.0310m/z	4,5-Dihydroorotic acid	Organic acids and derivatives
7.39_124.0146m/z	O-Phosphoethanolamine	Organic acids and derivatives
4.32_211.0506m/z	N-Acetylornithine	Organic acids and derivatives
0.64_174.0991n	N2-Acetyl-L-ornithine	Organic acids and derivatives
0.73_174.0628n	Formiminoglutamic acid	Organic acids and derivatives
0.62_174.0640n	Formimino-L-glutamic acid	Organic acids and derivatives
2.91_241.1183m/z	L-Saccharopine	Organic acids and derivatives
0.80_192.0259n	Isocitrate	Organic acids and derivatives
0.80_189.0625n	N-Acetyl-L-glutamic acid	Organic acids and derivatives
1.93_171.0288m/z	3-Dehydroquinate	Organic acids and derivatives
0.65_160.0603m/z	L-2-Aminoadipic acid	Organic acids and derivatives
1.35_144.0655m/z	Aminoadipic acid	Organic acids and derivatives
1.06_117.1024m/z	5-Aminopentanamide	Organic acids and derivatives
1.71_157.0971m/z	Theanine	Organic acids and derivatives
3.45_195.0767m/z	L-Theanine	Organic acids and derivatives
1.55_237.0846m/z	N-Acetyl-L-glutamate 5-semialdehyde	Organic acids and derivatives

2.58_72.0450m/z	Acrylamide	Organic acids and derivatives
5.99_886.4330m/z	Blasticidin S	Organic acids and derivatives
8.35_165.0873m/z	Glycyl-glycine	Organic acids and derivatives
3.29_153.1022m/z	Gly-Leu	Organic acids and derivatives
1.82_169.0971m/z	Glycylleucine	Organic acids and derivatives
3.50_172.0968m/z	Acetyl-DL-Leucine	Organic acids and derivatives
3.33_172.0968m/z	N-Acetylleucine	Organic acids and derivatives
2.91_440.2506m/z	N6-Acetyl-L-lysine	Organic acids and derivatives
1.45_320.1717m/z	Kyotorphin	Organic acids and derivatives
1.26_253.0825m/z	N2-Succinyl-L-ornithine	Organic acids and derivatives
1.94_132.0656m/z	Cis-4-Hydroxy-D-proline	Organic acids and derivatives
2.70_249.1233m/z	N-acetylphenylalanine	Organic acids and derivatives
3.99_207.0896n	N-Acetyl-L-phenylalanine	Organic acids and derivatives
6.08_237.1126m/z	N,N-Dimethylarginine	Organic acids and derivatives
0.57_188.1524n	N6,N6,N6-Trimethyl-L-lysine	Organic acids and derivatives
0.68_141.0425n	2-Aminomuconic acid semialdehyde	Organic acids and derivatives
0.97_112.0759m/z	1-Amino-1-cyclopentanecarboxylic acid	Organic acids and derivatives
1.22_145.0738n	Allysine	Organic acids and derivatives
1.52_146.0811m/z	L-2-Aminoadipate 6-semialdehyde	Organic acids and derivatives
2.99_229.0971m/z	Phenylacetylglutamine	Organic acids and derivatives
1.48_131.0816m/z	1-Pyrroline-5-carboxylic acid	Organic acids and derivatives
1.56_168.0291m/z	2-Amino-3-carboxymuconic acid semialdehyde	Organic acids and derivatives

5.73_256.0716m/z	D-Erythro-imidazole-glycerol-phosphate	Organic acids and derivatives
0.64_276.0954n	Gamma-Glutamylglutamic acid	Organic acids and derivatives
4.42_186.0763m/z	2-Keto-6-acetamidocaproate	Organic acids and derivatives
4.81_196.1083m/z	L-Histidine trimethylbetaine	Organic acids and derivatives
2.32_208.0607m/z	Hydroxyphenylacetylglycine	Organic acids and derivatives
4.85_176.0705m/z	Phenylacetylglycine	Organic acids and derivatives
8.33_362.9393m/z	Selenohomocysteine	Organic acids and derivatives
3.41_213.0659m/z	5-Hydroxyindoleacetylglycine	Organic acids and derivatives
2.05_517.2260m/z	Linatine	Organic acids and derivatives
0.71_343.1614m/z	4-Acetamido-2-aminobutanoic acid	Organic acids and derivatives
0.68_102.0552m/z	L-Azetidine 2-carboxylic acid	Organic acids and derivatives
3.32_158.0811m/z	4-Hydroxystachydrine	Organic acids and derivatives
2.94_539.2473m/z	Ceanothine B	Organic acids and derivatives
4.70_349.1758m/z	Enalaprilat	Organic acids and derivatives
1.72_158.1175m/z	Tranexamic acid	Organic acids and derivatives
0.70_188.1160n	N-Alpha-acetyllysine	Organic acids and derivatives
1.80_216.0983m/z	N-a-Acetylcitrulline	Organic acids and derivatives
3.46_232.1053n	4-(Glutamylamino) butanoate	Organic acids and derivatives
0.67_123.0321n	Isonicotinic acid	Organic acids and derivatives
5.63_414.1887m/z	AK toxin I	Organic acids and derivatives
0.75_144.0521n	N-Nitrosoproline	Organic acids and derivatives
3.66_385.2083m/z	Trandolaprilat	Organic acids and derivatives

3.07_376.1510m/z	Imidaprilat	Organic acids and derivatives
1.84_865.3703m/z	Quinaprilat	Organic acids and derivatives
1.61_397.1720m/z	Benazeprilat	Organic acids and derivatives
0.60_104.1072m/z	Choline	Organic nitrogen compounds
0.61_327.1780m/z	L-Histidinol	Organic nitrogen compounds
1.74_226.0953n	Porphobilinogen	Organic nitrogen compounds
1.54_138.0913m/z	2-Amino-1-Phenylethanol	Organic nitrogen compounds
7.58_122.0966m/z	N,N-Dimethylaniline	Organic nitrogen compounds
2.19_121.0744n	Tromethamine	Organic nitrogen compounds
0.69_159.1259n	METHACHOLINE	Organic nitrogen compounds
6.46_102.1279m/z	Triethylamine	Organic nitrogen compounds
4.65_225.0615m/z	Glucose	Organic oxygen compounds
0.64_191.0552m/z	Quinic acid	Organic oxygen compounds
1.66_174.0516n	Shikimic acid	Organic oxygen compounds
0.80_123.0442m/z	4-Hydroxybenzaldehyde	Organic oxygen compounds
0.73_195.0056m/z	Deoxyribose 1-phosphate	Organic oxygen compounds
1.38_369.1412m/z	Amylose	Organic oxygen compounds
4.35_227.1389m/z	6-Hydroxypseudooxynicotine	Organic oxygen compounds
5.35_230.0817m/z	Meglumine	Organic oxygen compounds
0.27_74.0607m/z	Aminoacetone	Organic oxygen compounds
5.81_550.2260m/z	Hygromycin B	Organic oxygen compounds
1.65_374.0183m/z	5-amino-1-(5-phospho-D-ribosyl)imidazole-4-carboxylate	Organic oxygen compounds

4.37_237.5677m/z	CMP-N-trimethyl-2-aminoethylphosphonate	Organic oxygen compounds
0.66_322.0440m/z	3'-CMP	Organic oxygen compounds
5.73_710.3019m/z	Natamycin	Organic oxygen compounds
0.75_400.1938m/z	Beta-D-Glucosamine	Organic oxygen compounds
0.76_98.0736n	3-propyl acrolein	Organic oxygen compounds
4.06_417.1906n	(E)-Casimiroedine	Organic oxygen compounds
3.53_238.1074m/z	Xanthoxylin	Organic oxygen compounds
1.05_195.0056m/z	1-Deoxy-D-xylulose 5-phosphate	Organic oxygen compounds
4.20_478.1924m/z	Validamycin A	Organic oxygen compounds
3.10_97.0289m/z	Furfural	Organic oxygen compounds
4.76_265.0607m/z	Necatorine	Organohalogen compounds
0.60_140.9033m/z	Chloroform	Organohalogen compounds
0.76_167.0199m/z	Uric acid	Organoheterocyclic compounds
2.24_160.0756m/z	Indoleacetaldehyde	Organoheterocyclic compounds
4.20_201.1021m/z	Indole-3-acetaldehyde	Organoheterocyclic compounds
1.27_220.0848n	5-Hydroxy-L-tryptophan	Organoheterocyclic compounds
0.71_138.0430n	Urocanic acid	Organoheterocyclic compounds
3.26_289.1364m/z	Alanyltryptophan	Organoheterocyclic compounds
0.74_183.0531n	4-Pyridoxic acid	Organoheterocyclic compounds
4.63_144.0807m/z	Tryptophanol	Organoheterocyclic compounds
3.40_263.1036m/z	N-Acetylserotonin	Organoheterocyclic compounds
4.05_188.0344m/z	Kynurenic acid	Organoheterocyclic compounds

0.81_86.0969m/z	Piperidine	Organoheterocyclic compounds
3.06_213.0658m/z	Isophenoxazine	Organoheterocyclic compounds
6.30_208.0732n	6-Methoxymellein	Organoheterocyclic compounds
0.79_326.1226n	6,7-Dimethyl-8-(1-D-ribityl)lumazine	Organoheterocyclic compounds
0.72_93.0451m/z	Imidazole-4-acetaldehyde	Organoheterocyclic compounds
0.68_171.0399m/z	Hydantoin-5-propionic acid	Organoheterocyclic compounds
4.88_194.0464m/z	5,6-Dihydroxyindole	Organoheterocyclic compounds
3.38_190.0501m/z	5-Hydroxyindoleacetic acid	Organoheterocyclic compounds
4.67_173.1073m/z	5-Methoxytryptamine	Organoheterocyclic compounds
2.33_625.2835m/z	D-Urobilinogen	Organoheterocyclic compounds
8.23_144.0441m/z	3-Methyldioxyindole	Organoheterocyclic compounds
4.85_130.0651m/z	Isoquinoline	Organoheterocyclic compounds
2.42_351.1308m/z	FAPy-adenine	Organoheterocyclic compounds
2.64_197.1285n	4-ethylamino-6-isopropylamino-1,3,5-triazin-2-ol	Organoheterocyclic compounds
5.69_271.1662m/z	Epsilon-caprolactam	Organoheterocyclic compounds
4.10_277.0841m/z	Phenobarbital	Organoheterocyclic compounds
3.52_131.0723n	3-Methylindole	Organoheterocyclic compounds
3.74_145.0515n	1H-Indole-3-carboxaldehyde	Organoheterocyclic compounds
3.97_252.1349m/z	Arenaine	Organoheterocyclic compounds
4.16_148.0392m/z	Isatin	Organoheterocyclic compounds
6.81_298.3100m/z	Tridemorph	Organoheterocyclic compounds
5.41_196.0872m/z	Debrisoquine	Organoheterocyclic compounds

5.64_662.3053m/z	Carbofuran	Organoheterocyclic compounds
3.52_203.0818m/z	Nirvanol	Organoheterocyclic compounds
0.71_70.0657m/z	1-Pyrroline	Organoheterocyclic compounds
6.09_257.1502m/z	1,4'-Bipiperidine-1'-carboxylic acid	Organoheterocyclic compounds
6.18_239.1282m/z	Neocnidilide	Organoheterocyclic compounds
3.49_85.0289m/z	2(5H)-Furanone	Organoheterocyclic compounds
3.43_152.0706m/z	2-Descarboxy-cyclo-dopa	Organoheterocyclic compounds
0.61_166.0862m/z	5-(3-Pyridyl)-2-hydroxytetrahydrofuran	Organoheterocyclic compounds
2.99_258.1447m/z	Alpha-[3-[(Hydroxymethyl)nitrosoamino]propyl]-3-pyridinemethanol	Organoheterocyclic compounds
3.65_144.0444m/z	Indole-3-carboxylic acid	Organoheterocyclic compounds
2.24_118.0653m/z	Indole	Organoheterocyclic compounds
2.92_169.0756m/z	Norharman	Organoheterocyclic compounds
1.53_99.0446m/z	2-Furanmethanol	Organoheterocyclic compounds
4.02_478.2303m/z	Fumitremorgin B	Organoheterocyclic compounds
1.52_440.2142m/z	Paliperidone	Organoheterocyclic compounds
0.74_78.0142n	Dimethyl sulfoxide	Organosulfur compounds
0.74_111.0919m/z	L-Lysine	Peptides
0.60_175.1188m/z	L-Arginine	Peptides
0.78_145.0605m/z	L-Glutamine	Peptides
1.75_88.0398m/z	L-Serine	Peptides
0.76_97.0764m/z	Ornithine	Peptides
2.24_203.0818m/z	L-Tryptophan	Peptides
1.66_164.0705m/z	L-Phenylalanine	Peptides
0.97_181.0729n	L-Tyrosine	Peptides
0.51_156.0767m/z	L-Histidine	Peptides
2.30_344.1823m/z	L-Proline	Peptides
0.73_117.0791n	L-Valine	Peptides

0.79_84.0449m/z	L-Threonine	Peptides
0.60_146.0445m/z	L-Glutamate	Peptides
0.51_225.0986m/z	Carnosine	Peptides
0.81_131.0947n	L-Isoleucine	Peptides
1.64_120.0808m/z	Tyramine	Peptides
6.08_188.9505m/z	3-Sulfinoalanine	Peptides
0.62_159.0763m/z	D-Ala-D-Ala	Peptides
0.78_176.0553m/z	4-Hydroxyproline	Peptides
1.40_285.1202m/z	Anserine	Peptides
2.15_171.0763m/z	Pyroglutamic acid	Peptides
2.24_176.0377m/z	N-Formylmethionine	Peptides
6.15_132.0576n	Cinnamaldehyde	Phenylpropanoids
5.92_385.0917m/z	Scopoletin	Phenylpropanoids
0.79_164.0473n	2-Hydroxycinnamic acid	Phenylpropanoids
0.51_291.0507m/z	Isopimpinellin	Phenylpropanoids
5.59_163.0389m/z	UMBELLIFERONE	Phenylpropanoids
5.74_134.0734n	Chavicol	Phenylpropanoids
4.02_725.3465m/z	Secoisolariciresinol	Phenylpropanoids
1.65_148.0523n	Dihydrocoumarin	Phenylpropanoids and polyketides
2.52_192.1018m/z	Hydrocinnamic acid	Phenylpropanoids and polyketides
5.93_891.4114m/z	Rifabutin	Phenylpropanoids and polyketides
6.37_226.1587m/z	2,4-Diphenyl-1-butene	Phenylpropanoids and polyketides
3.49_269.1141m/z	Hypoglycin B	Phytotoxins
1.54_141.0791n	Hypoglycin	Phytotoxins
4.02_283.1654m/z	L-Hypoglycin A	Phytotoxins
5.95_546.2676m/z	Crocin 4	PK Polyketides
6.88_931.5921m/z	Sirolimus	PK Polyketides
5.30_465.1036m/z	Diospyrin	PK Polyketides
5.31_215.1066m/z	4,4'-dihydroxybibenzyl	PK Polyketides
0.68_144.0655m/z	Maltol	Polyketides
6.29_318.2400m/z	4-Oxoretinol	PR Prenol Lipids
5.87_343.1870m/z	(S)-[8]-Gingerol	Skimate / acetate-malonate pathway derived compounds
6.12_193.0860m/z	Zingerone	Skimate / acetate-malonate pathway derived compounds
7.83_537.5121n	N-Palmitoylsphingosine	SP Sphingolipids
6.54_340.2628m/z	Sphinganine	SP Sphingolipids
5.28_524.2860m/z	Psychosine sulfate	SP Sphingolipids

6.20_371.3265m/z	Dihydroceramide	SP Sphingolipids
4.86_218.2115m/z	Dodecanoic acid	ST Sterol Lipids
4.13_528.3040m/z	Lithocholyltaurine	ST Sterol Lipids
1.51_509.2021m/z	Estriol-16-Glucuronide	ST Sterol Lipids
5.39_463.2336m/z	Hydrocortisone succinate	ST Sterol Lipids
5.69_315.1336m/z	Estrone	Steroids
5.98_803.3973m/z	Cortisone acetate	Steroids
4.33_629.2806m/z	Ouabain	Steroids
6.48_994.6423m/z	Glycocholic acid	Steroids
6.33_391.2849m/z	Deoxycholic acid	Steroids
5.79_536.2616m/z	Taurocholic acid	Steroids
5.36_306.2062m/z	Estriol	Steroids
5.23_747.4271m/z	Digitoxin	Steroids
5.91_229.1221m/z	Abscisic acid	Terpenoids
0.77_519.2048m/z	Gossypol	Terpenoids
2.40_495.2212m/z	Glaucarubin	Terpenoids
2.66_484.2761m/z	Withanolide	Terpenoids
5.97_627.3294m/z	Agavoside A	Terpenoids
6.12_1029.5264m/z	Protodioscin	Terpenoids
6.13_1031.5409m/z	Musennin	Terpenoids
5.15_633.2946m/z	Soyasaponin A1	Terpenoids
6.11_942.5194n	Soyasaponin I	Terpenoids
0.95_284.1968m/z	(2R,6S,7S,10Z)-beta-Santala-3(15),10-dien-12-ol	Terpenoids
6.13_195.1016m/z	(+)-(S)-Carvone	Terpenoids
3.13_712.3301n	Convalloside	Terpenoids
0.62_123.0554m/z	Niacinamide	Vitamins and Cofactors
1.92_212.0557m/z	Pyridoxal	Vitamins and Cofactors
3.39_500.2715m/z	L-erythro-tetrahydrobiopterin	Vitamins and Cofactors
2.37_440.1323m/z	Folic acid	Vitamins and Cofactors
4.49_200.0920m/z	D-Pantothenic acid	Vitamins and Cofactors
1.84_219.1107n	Pantothenic Acid	Vitamins and Cofactors
